# Repair of bony lateral skull base defects equal to or larger than 10 mm by extracorporeally sewed unit-sandwich graft

**DOI:** 10.1007/s00405-018-5039-8

**Published:** 2018-06-23

**Authors:** Shabbir Indorewala, Gaurav Nemade, Abuzar Indorewala, Gauri Mahajan

**Affiliations:** Indorewala ENT Hospital and Research Centre, Gaikwad Nagar, Behind Mahamarg Bus Stand, Nashik, Maharashtra 422002 India

**Keywords:** Brain herniation, Dural herniation, Herniation of cranial contents, CSF leak, Large skull base defect, Fascia-bone-fascia sandwich, Multi-layered graft, Unit-sandwich graft

## Abstract

**Objective:**

To see effectiveness of the senior author’s repair technique for repair of large (equal to or larger than 10 mm) bony lateral skull base defects.

**Study design:**

Retrospective.

**Settings:**

Secondary/tertiary care center.

**Methods:**

We performed retrospective review of 9 surgeries done in our institution between January 2010 and December 2013 for repair of large lateral bony skull base defects. We defined skull base defects extra-cranially and repaired them intra-cranially. We made an extracorporeal sandwich of autologous fascia-bone-fascia (fascia lata and nasal septal bone) and sewed it together to make it into a unit-sandwich graft. This extracorporeally sewed unit-sandwich graft was then inserted to close the large skull base defects either via (1) a cranial slit-window, or (2) the skull base defect itself. Since skull base is bony, bony repair is preferred. Bone plates that are easily available for skull base repair are calvarial and nasal septal bone. Occasionally, harvest of split calvarial bone carries risk of major complications. We preferred nasal septal bone. Harvesting of septal bone even in children using a posterior incision should not disturb the cartilage growth centers.

**Results:**

All nine patients were operated by this technique. We had four patients with cerebrospinal fluid leak, and five patients with brain herniation. All these patients had complete reversal of herniation of cranial contents and cessation of cerebrospinal fluid leak. On imaging, in 6 cases the bone graft remained in original intended position after 12 months of surgery. The bone graft was not identifiable in 3 cases.

**Conclusion:**

The senior author’s technique using autologous multi-layered graft is simple to master, repeatable and very effective.

## Introduction

Large lateral bony skull base defect (defects larger than 10 mm diameter) must be repaired. If these skull base defects are not repaired’, they present later in life with either dural herniation or brain herniation or fungus cerebri; all of them associated with or without cerebrospinal fluid (CSF) leak. These patients may remain asymptomatic for some time or may present with any one or more symptoms like recurrent meningitis, CSF otorrhoea, CSF otorhinorrhea, CSF rhinorrhea, hearing loss, recurrent ‘secretory otitis media’, mass or polyp in open mastoid cavity or nasal cavity. Diagnosis of these defects is sometimes obvious and sometimes very difficult. It depends on type and presentation. High index of suspicion, proper utilization, and interpretation of diagnostic tools are required to diagnose this condition. Repairs of large skull base defects have posed as a challenge to surgeons for years. The use of xenograft (canine) dura to repair a postoperative CSF leak with good results was first described in 1903 by Canfield [1]. Dandy later established the use of autologous tissue for the repair of CSF otorrhoea [1]. Many authors have suggested the use of fibrin glue, bone cement, and autologous tissue, alone or in multiple combinations, to guarantee effective closure [2–7]. No ideal procedure and repair technique exists, but there is a general agreement that multi-layer closure significantly reduces the recurrence rate [2]. A commonly used and effective method of multi-layer closure is the fascia-bone-fascia technique [8, 9]. Critics of this method, however, point to reports of bone graft migration or resorption [5]. To prevent migration and to ease the surgical exercise of repair of the defects, we have modified the technique. We harvest fascia lata and nasal septum. Out of these grafts, we make an extracorporeal sandwich of fascia-bone-fascia and sew them together to make them into a unit-sandwich. This unit-sandwich has resulted in great ease of insertion of the three-layered graft, complete closure of bony defect with good clinical outcomes and has maintained proper graft position evident on postoperative imaging. The strategy and modification of skull base defect repair practiced at our institution is distinctive. And to our knowledge, this is the first reported series with such modification. It is our goal to provide an option for the surgical repair of large skull base defects. We find this strategy and modification simple and effective.

## Materials and methods

We performed a retrospective review of cases done in our institution using the below described method of repair for large lateral skull base defects for the period between January 2010 and December 2013. Strict inclusion criteria were: (1) cases in which preoperative and 12 months postoperative radiologic images were available, (2) had a minimum of 24 months follow-up. We had a total of 9 cases (Table 1).

**Table 1:** Summary of cases

SN	Name	Age/sex	MYS	Site	Etiology	CSF leak	DD	BD	HR	IW	F/U	Bone graft after 12 months post OP imaging
1	SP	25/F	Mar-13	MCF	Surgery	N	NIL	13	Y	SW	30	Identifiable and in place
2	AP	43/M	Apr-11	MCF	Surgery	Y	3 mm	15	N	SW	58	Identifiable and in place
3	KR	40/F	Jul-12	MCF	Spontaneous	Y	2 mm	12	Y	SW	26	Identifiable and in place
4	SF	27/F	Oct-10	MCF	Cholesteatoma	N	NIL	10	Y	SW	52	Not identifiable
5	PS	13/F	Jul-10	MCF	Cholesteatoma	Y	1 mm	12	N	SW	37	Identifiable and in place
6	BY	45/M	Jul-13	MCF	Cholesteatoma	N	NIL	13	Y	SW	28	Not identifiable
7	QA	5/M	Jan-11	MCF	Congenital	N	NIL	10	N	SW	32	Identifiable and in place
8	SJ	56/F	Aug-11	PCF	Spontaneous	Y	2 mm	10	Y	DW	39	Not identifiable
9	KB	7/M	Nov-11	MCF	Cholesteatoma	N	NIL	12	N	SW	43	Identifiable and in place

All cases had complete reversal of hernia of cranial contents. No case had any kind of morbidity. Mean follow-up: 37 months

*MYS* month and year of surgery, *MCF* middle cranial fossa, *PCF* posterior cranial fossa, *DD* dural defect, *BD* bony defect, *HR* brain herniation, *IW* insertion window, *F/U* follow up in months after surgery, *SW* slit window, *DW* defect window

### Technique

#### Extra-cranial definition of skull base defect

For repair of large lateral skull base defects (defects larger than 10 mm diameter), we performed mastoidectomy or revision mastoidectomy and defined extra-cranially circumferential margin of skull base defects. Herniated cranial contents were attempted and pushed back in cranial cavity. If there was fungus cerebrii, it was excised using a bipolar cautery. Dura was then lifted circumferentially beyond the margins of bony defects by at least 3–5 mm.

#### Harvest of graft materials

We harvested autologous bony nasal septum through Killian’s incision. The size of the bone harvested is important. It is 3–5 mm longer than the distance between the medial edge of the skull base defect and the outer surface of the squamous temporal, and 3–5 broader than anterior–posterior distance of the skull base defect. We harvest autologous fascia lata from lateral aspect of thigh, 100 mm proximal to the knee joint. The size of the fascia lata graft is big enough to cover the bone graft on both surfaces.

#### Preparation of extracorporeal fascia-bone-fascia unit-sandwich

**Fig. 1:**
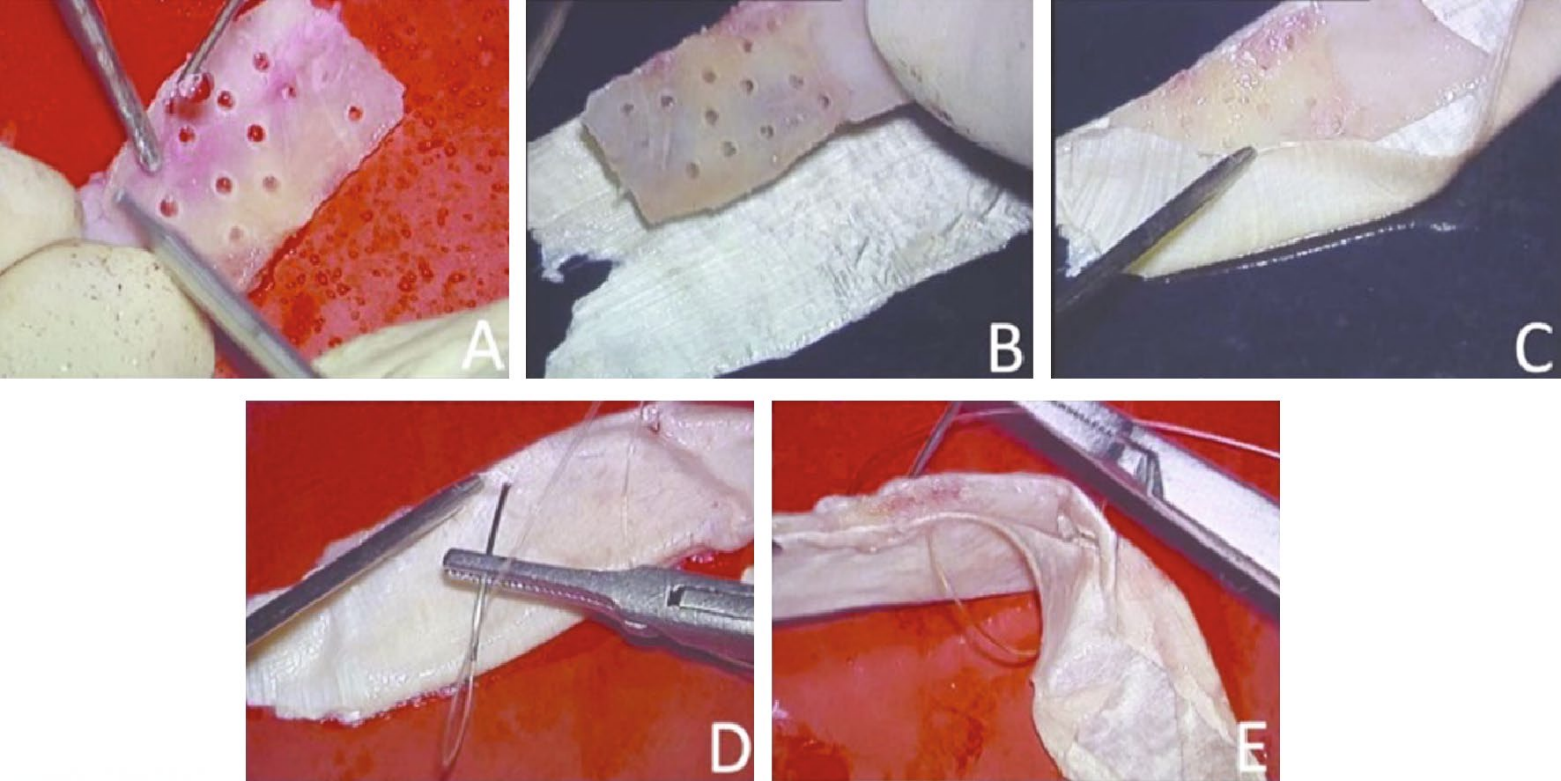
**a** Under continuous saline irrigation multiple random holes are made in a sized harvested nasal septal bone graft. **b** The perforated septal bone is placed on fascia lata. **c** The fascia lata is turned over so as to wrap the perforated septal bone on both surfaces. **d, e** The sandwich is sutured through and through to achieve the unit-sandwich graft

Using otologic drill and a 0.5 mm coarse diamond burr, we drill multiple random holes in the bone plate approximately 2 mm away from each other. Multiple holes drilled in the septal bone is similar to holes drilled in septal bone during extracorporeal septoplasty [10]. Multiple holes made in the bony plate allow passing of needle and suture material so as to make a unit-sandwich. This bone plate with multiple random holes is now placed on fascia lata, and the remaining part of the fascia lata (fascia lata is twice the size of bone plate) is turned over to make a sandwich, with fascia lata on both surfaces of the bone plate (Fig. 1). We use absorbable suture (undyed monofilament polyglecaprone 25, Monocryl, Ethicon) to sew the sandwich. The suture passes through the fascia on one surface of the perforated bone plate, and through the hole in the bone plate and out through the fascia on the other surface of the bone plate. The continuous sewing process is repeated till all the holes in the bone plate are consumed and the two ends of the suture are tied together. This result in an autologous sandwich of fascia-bone-fascia sewed together into a unit by a continuous suture passing through and through its component layers.

#### Making of cranial slit-window

**Fig. 2:**
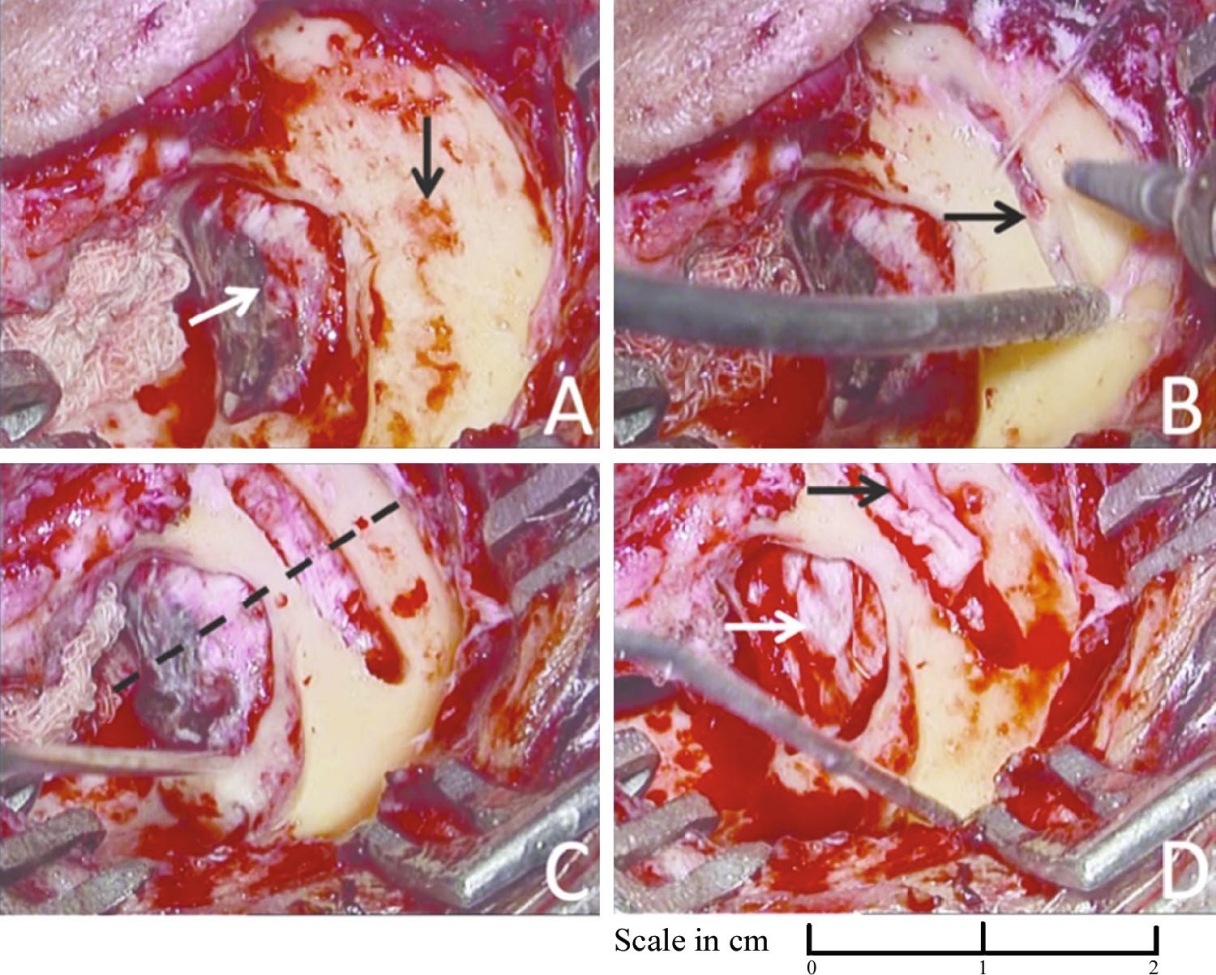
**a** Open mastoid cavity with circumferentially delineated large skull base defect (white arrow). Squamous surface of temporal bone immediately adjacent of the skull base defect is clearly exposed (black arrow). **b** Cranial slit-window is made (black arrow). **c** The slit-window is completed. Note (1) the perpendicular bisector of the slit-window passes through the anterior–posterior centre of the skull base defect (dashed line), and (2) the length of the cranial slit-window is longer by 2–5 mm than the anterior–posterior length of the skull base defect. **d** Photograph showing unit-sandwich graft inserted through the slit just before its final placement. Note that the lateral end of the unit-sandwich graft seals the slit-window (black arrow) and the surface of the unit-sandwich seals the skull base defect (white arrow)

**Fig. 3:**
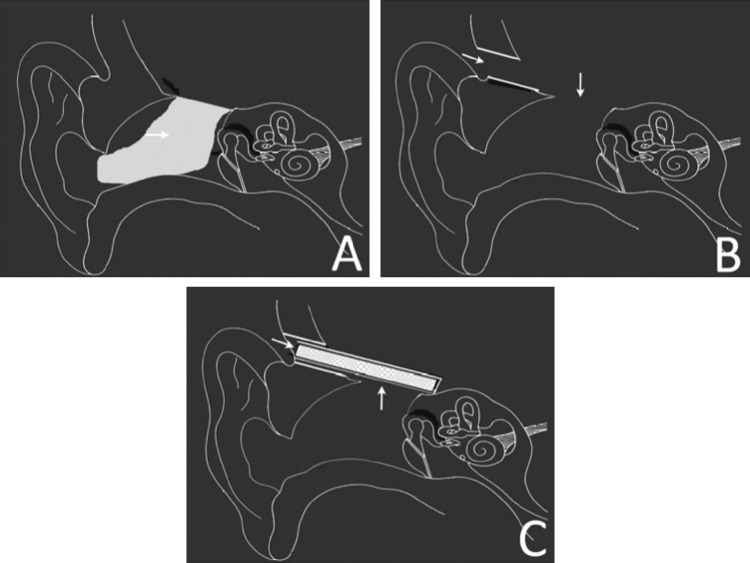
Diagrammatic representation of lateral skull base defect repair using unit-sandwich graft and cranial slit-window. **a** Large tegmen defect with fungus cerebrii (horizontal arrow) in open mastoid cavity and external auditory canal. **b** Cranial slit-window (horizontal arrow) is made on the squamous surface of the temporal bone as close to the tegmen as possible. Fungus cerebrii is excised. Tegmen defect (vertical arrow) is clearly seen. **c** Unit-sandwich graft is placed in position. It is composed of three layers, sutured together. The outer end of the graft seals the slit-window (horizontal window) and the surface of the unit-sandwich graft seals the tegmen defect (vertical arrow). The unit-sandwich graft is placed in intracranial-extradural plane. It provides a durable barrier between the extra-cranial and intra-cranial compartments, preventing herniation of cranial contents and stabilizing the bone graft from migration

For lateral skull base defects, we preferred a cranial insertion slit-window on the squamous part of the temporal bone which is similar to the mini-craniotomy described by Sanna et al. [11]. We make slit-window using coarse diamond burr. The size of the burr is a couple of millimeters bigger than the thickness of the unit-sandwich to be inserted. The insertion slit is just broad and long enough for the unit-sandwich to be pushed in. The cranial slit-window is positioned on the squamous part of the temporal bone in such a way that the perpendicular bisector of the slit-window passes through the anterior–posterior centre of the skull base defect (Fig. 2). The window is made as close to tegmen as possible (Fig. 3) to reduce brain retraction and to avoid intra-cranial dead space under the unit-sandwich graft. Once the slit-window is created, the dura is lifted from the superior surface of the temporal bone through the slit so as to expose the skull base defect through the slit.

#### Repair of the skull base defect

The unit-sandwich graft is now inserted through the slit-window in the intracranial-extradural plane in such a way that the surface of the unit-sandwich closes the skull base defect. In this way, the unit-sandwich graft lies flat on the superior surface of the tegmen and the petrous with its outer edge closing the slit-window (Figs. 2d, 3c). The advantage of the cranial slit-window is obvious. It allowed us to insert the unit-sandwich graft slightly bigger than the skull base defect, thus closing the skull base defect circumferentially. The outer (lateral) end of the unit-sandwich graft closed the slit-window. Of the 9 lateral skull base defects, 8 were middle cranial fossa defects and one was posterior cranial fossa defect. For insertion of the unit-sandwich graft, we used skull base defects in 1 case and slit-windows in 8 cases. The follow-up period was at least 24 months (Mean follow-up 37 months). Table 1 shows various etiologies for skull base defects. Surgery was responsible in 2 cases, 2 were spontaneous, 4 were due to cholesteatoma, and 1 was of congenital origin. In our present series, we did not use fibrin glue.

## Results

**Fig. 4:**
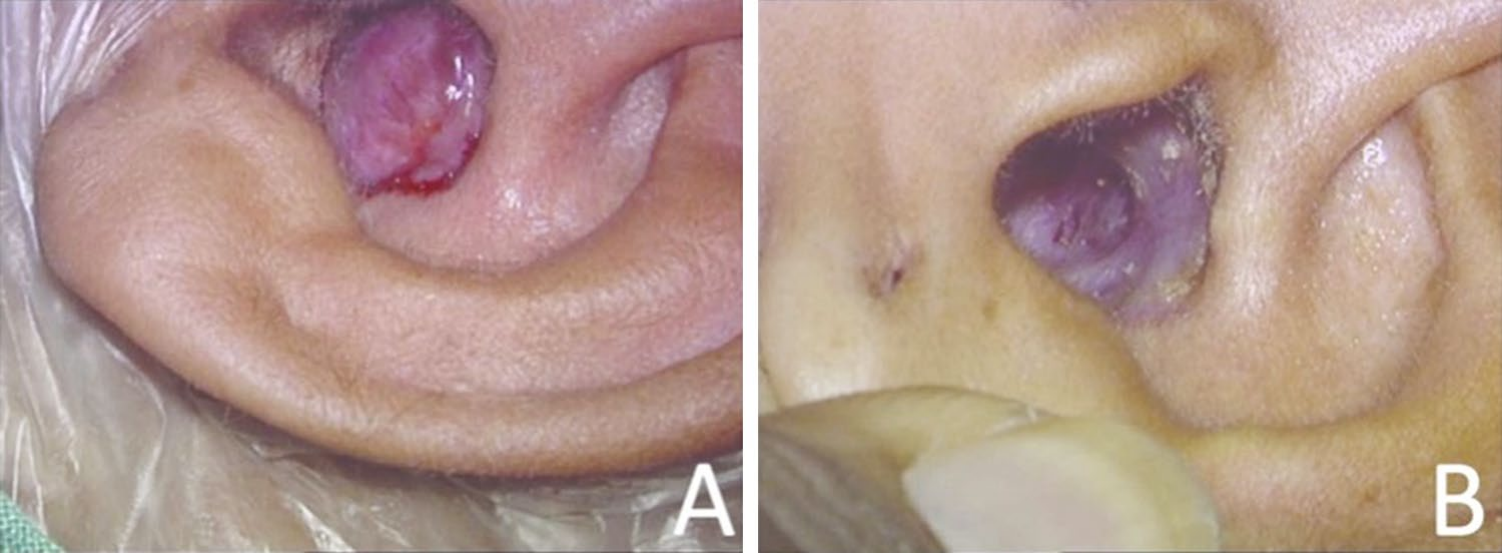
**a** Preoperative view of brain herniation seen in the auditory canal. **b** Postoperative view of the same patient after 2 years of surgery. Dry, clean, open mastoid cavity with (repaired) skull base is clearly visible without any sagging or herniation

**Fig. 5:**
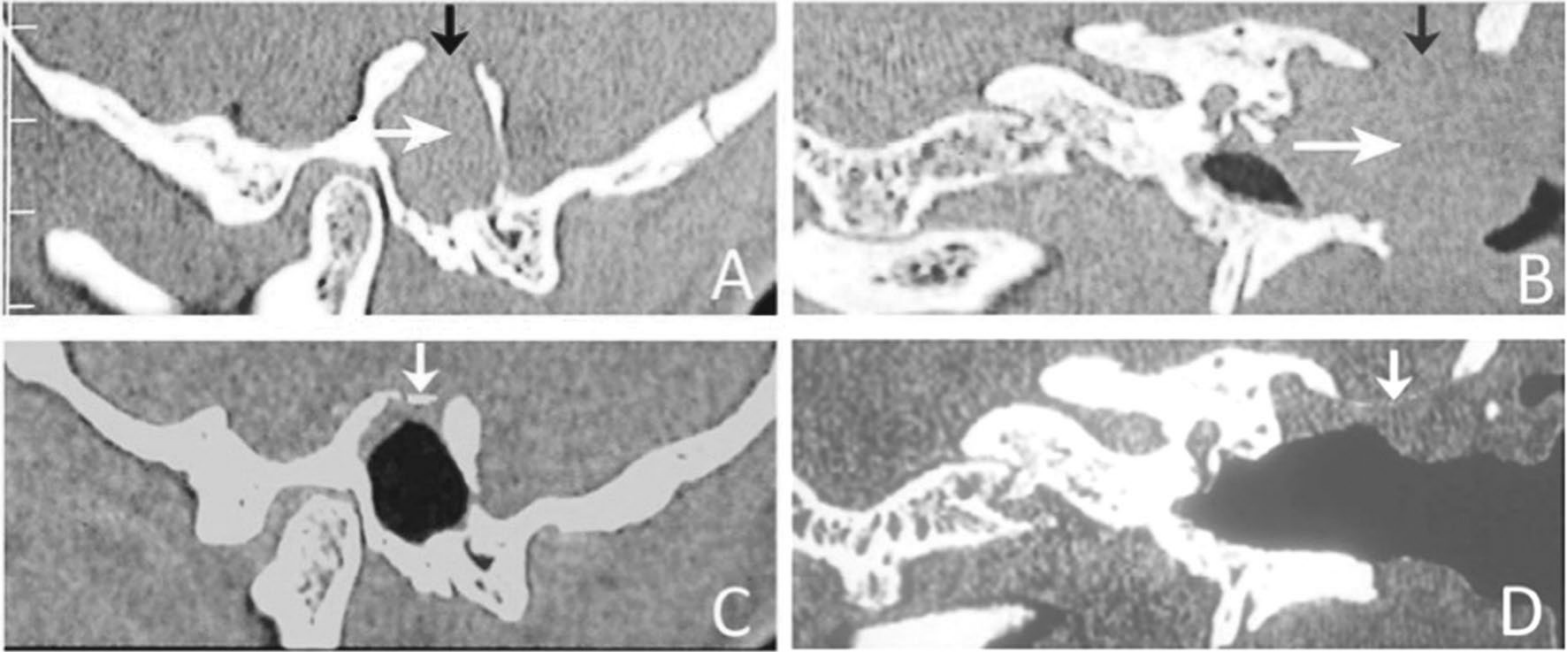
Preoperative (**a, b**) and 1-year postoperative (**c, d**) coronal and parasagittal computed tomography scan images passing through the skull base defect. **a, b** Note the large skull base defect (black arrow) and a large herniation of the cranial contents (white arrow) in the external auditory canal and the open mastoid cavity. **c, d** Note the clearly defined bone shadow (white arrow) of the unit-sandwich graft. The bone graft has maintained its original intended position

All cases had complete reversal of herniation of the contents of the cranial cavity with complete cessation of CSF leak after placement of the unit-sandwich graft in its final destination. These patients were followed-up for a minimum period of 24 months. All cases maintained complete reversal of herniation of contents of cranial cavity (Fig. 4). No case had recurrence of CSF leak. Imaging was done at 12 months postoperative period (Fig. 5). The bone graft did not show resorption in 6 cases, and was seen in its original intended position. In 3 cases, bone graft was not identifiable (Table 1). We had no case of meningitis, intra-cranial abscess or any other morbidities or mortality.

## Discussion

### Surgical approach

Sub-temporal approach was used to repair large tegmen defects [12]. The approach had advantages like (1) better control of the anterior part of the roof of the petrous bone, (2) preservation of hearing by avoiding manipulation of the ossicular chain, and (3) reduced risk of contamination by a possibly infected middle ear cavity. However, due to greater invasiveness and morbidity of sub-temporal approach, some authors suggest reserving it for spontaneous meningoencephaloceles with preserved hearing [13]. Other commonly used approaches include a middle cranial fossa craniotomy alone [14], a mini-middle cranial fossa approach [15], a transmastoid approach [16], and a combined middle cranial fossa-transmastoid approach [17]. The choice of surgical approach depends on advantages and disadvantages of each approach with regard to the etiology of the defect, the site and the extent of bony defect, herniation of cranial contents, the type and the degree of hearing loss, presence of chronic infection in the middle ear, and by the intraoperative finding of active CSF leakage and also the surgeon’s personal experience and comfort level with the approach in question [13] (Table 2).

**Table 2:** Review of literature

Author	Year	No. of cases	Etiology	Approach	Materials	F/U in months	Results in %	Comments
Adkins et al.	1983	6	COM 2, Iatrogenic 2, Congenital 1, Posttraumatic 1	Combined	Autogenous graft	X	X	Minicraniotomy facilitates precise extradural, intracranial placement of graft over tegmen defect, avoiding morbidity and potential complications of full MCF approach
Golding-Wood et al.	1991	4	X	Subtemporal	Bone, Fascia	X	X	For surgical repair of tegmen defects with brain herniation, placing of bone enveloped by fascia via subtemporal approach is preferred
Lundy et al.	1996	19	Spontaneous 11, COM 4, Iatrogenic 2, Posttraumatic 2	Combined 16, MCF 2, TM 1	Bone, Fascia	31	100	Repair was accomplished in one stage in all cases by placing fascia-bone-fascia graft extradurally (multilayer technique)
Mosnier et al.	2000	15	COM 9, CH 5, Iatrogenic 1	Combined 11, MCF 4	Bone, Fascia	24	100	Extradural repair with fascia and bone using combined MCF-TM approach along with resection of the herniated part should be done in one or 2 stages, when necessary
Dutt et al.	2001	4	Spontaneous 4	MCF	Autologous bone pate, fibrin glue, temporalis fascia	12–36	100	MCF approach is more effective than TM approach for tegmen defects with additional advantage of hearing preservation. Bone pate with soft tissue and glue achieve secure sealing of tegmen defects especially multiple without any risk of migration
Savva et al.	2003	92	X	X	Bone wax, free muscle, fascia, allogenic material(fibrin glue)	24	100 in multilayer closure and 75.4 in single layer closure	No additional benefit of using fibrin glue with primary closure. Multilayer closure technique is recommended
Nahas et al.	2008	15	Spontaneous 15	Combined	HAC, calvarial bone, fascia	X	100	Combined approach has advantage of optimal access to the tegmen defect. Repair of both dural and bony defects is necessary. HAC with bone grafting is helpful in filling small cortical defects
Sanna et al.	2009	133	Iatrogenic 45.9%, Spontaneous 24.8%, COM 21.8%, Posttraumatic 7.5%	TM 27.8%, MCF 27.8% Combined 3%, EO 41.4%	X	Mean 38.4	X	Choice of approach must be based on the location and size of the herniated tissue, audiological status, concomitant pathology
Ota et al.	2010	3	Iatrogenic	TM	HAC, fat, fibrin glue	X	100	HAC (using multilayer technique) is effective biomaterial for repair of refractory CSF leak due to opening of air cells in deeper surgical fields

*COM* chronic otitis media, *TM* transmastoid, *MCF* middle cranial fossa, *HAC* hydroxy appetite cement, *MEO* middle ear obliteration with blind sac closure of the external auditory canal, *CH* cholesteatoma

In Nahas et al. series, mastoidectomy was performed to confirm the presence and the location of herniation of cranial contents and an associated CSF leak. Then a mini-craniotomy (3 × 4 cm) of the middle fossa was located with inferior edge at 5–10 mm of the tegmen to optimize visualization of the defects while minimizing the temporal lobe retraction [18].

We performed mastoidectomy or revision mastoidectomy and defined extra-cranially circumferential margin of lateral skull base defects similar to Nahas’ method. Instead of mini-craniotomy, as done by Nahas, we made a cranial slit-window which is similar to the mini-craniotomy described by Sanna et al. [11]. This slit-window has three advantages over mini-craniotomy. Slit-window is easy to make, it does not need closure after the procedure as the lateral end of the unit-sandwich graft seals the slit-window and retraction of the temporal lobe is minimal as the slit-window is made close to tegmen. On the other hand, mini-craniotomy is cumbersome to make, special drills are required to make, it needs to be closed after reduction and repair of herniation of cranial contents, and it needs some amount of temporal lobe retraction.

### Repair materials

Homografts and xenografts share common drawbacks of infection, availability, difficulty in harvesting, resorption, instability and lack of integration [19]. Synthetic materials like bone substitutes, cements, ceramics, metals, polymers, acrylics and their composites have drawbacks like infection, migration or extrusion [20–22]. It is now nearly agreed upon that autologous grafts are the most preferred material [23]. Because of high failure rates using other methods, autologous multi-layered sandwich techniques are recommended [19]. Repair material preferably should have a soft and a hard component. The soft component on the cranial surface of the hard component is required to repair the dural defects and to stop CSF leak. Soft component is also required on the extra-cranial surface of the hard component so as to protect the hard component and to facilitate mucosal or epithelial lining on the external surface. Hard component is required to hold the cranial contents in place so as to prevent herniation of the cranial contents.

Since skull base is bony, bony repair is preferred. It is better to avoid cartilage (auricular cavum cartilage or nasal septal cartilage) as repair material, particularly in children whose cartilages are quite soft. Bone plates that are easily available for skull base repair are calvarial bone and nasal septal bone. Occasionally, harvest of split calvarial bone carry risk of intra-cerebral hematoma, subarachnoid hemorrhage, dural tears and CSF leaks [24]. It is time-consuming and the donor site is biomechanically less stable [25]. Splitting of calvarial bone requires experience, as sometimes the bone cracks into several pieces. We thus prefer nasal septal bone. Harvesting of septal bone even in children using a posterior incision should not disturb the cartilage growth centers. It is devoid of major complications and harvested quickly.

Harvest of fascia lata needs a separate incision and does cause a minor additional morbidity and an additional scar on thigh. It however has an advantage of getting a large graft (10 mm × 20 mm or more) through a small (8 mm) mobile skin incision. Harvest of nasal septal graft may result in inadvertent septal perforation.

### Preparation of extracorporeal fascia-bone-fascia unit-sandwich

Placing the component layers of tissues (one layer after another layer) under brain in the intracranial-extradural plane is difficult and time-consuming. After placing a soft tissue layer without folds and wrinkles in that difficult situation, attempting to place a bony component disturbs the previously placed soft tissue layer. In the same way while attempting to place the outer soft tissue layer, the bony component gets displaced and disturbed. To make placement of these three layers easy and accurate, we make an extracorporeal sandwich of the three layers and sew all of them together into a unit as described in technique above. Extracorporeal suturing is easy and more accurate. Without disturbing component layers, extracorporeally made unit-sandwich graft is easy to handle and can be placed in its final destination (intracranial-extradural plane) quickly and effectively through a slit-window. We do not need to make even a ‘mini’ craniotomy.

### Making of cranial slit-window

The cranial slit-window is made close to tegmen (Fig. 3b). Slit-window made close to tegmen needs minimal brain retraction and also reduces dead space between the graft and the skull base. The slit-window is easy to make with a 5 mm coarse diamond burr with otologic drill. The slit itself does not need closure as the lateral end of unit-sandwich seals the slit-window. All this makes surgery quick and simple. Making a cranial slit-window has an advantage that it can take care of anterior (attic), mid or posterior (antral) middle fossa bony defects. We have managed the largest defect of 15 mm. However depending on graft size, even larger bony defects can be managed. Making of a cranial slit-window does not need exploration of attic or middle ear, thus surgical manipulation of the ossicular chain is not required.

### Long-term results

A common problem with previous technique was migration of autologous bone graft and resorption [5]. By making an extracorporeally sewed unit-sandwich, the bone gets anchored to the soft tissues. Soft tissues do not migrate. The bone, anchored with sutures to soft tissue, also does not migrate. Follow-up computed tomography of temporal bone images in multiple planes after 12 months of surgery confirmed that the bone grafts to have remained in their original intended position with good reconstruction of the lateral skull base (Fig. 5). In three cases, bone grafts were not identifiable. This could be due to the fact that the bone grafts used were rather thin and the grafts could not be picked up on computed tomography. Or it could be that the thin bone grafts get resorbed. Further studies are needed to resolve this issue. Our minimum postoperative follow-up of 24 months revealed satisfactory clinical outcomes with no evidence of recurrent cranial herniation or cerebrospinal fluid leak.

## Conclusion

There are many alternatives described in the literature for repairing large skull base defects.

Advancements in materials and approaches over years have allowed skull base surgeons to enormously improve the outcomes of large skull base defect repairs. However, agreement on the best method of repair is lacking. Regardless of the reconstruction methods used, the primary goals of any large skull base defect repair should focus on: (1) providing a durable (long-term) barrier between the extra-cranial and intra-cranial compartments, so as to prevent herniation of cranial contents, (2) attaining a durable watertight seal, (3) should be easy to execute and (4) should be repeatable. Based on our experience, we believe that the use of autologous multi-layered extracorporeally made unit-sandwich graft and the use of slit-window provide an effective combination for repair of large lateral skull base defects. It seems to attain all the above-mentioned goals of repair of large skull base defects. The technique utilizes multiple layers of autologous tissues to repair the defect, re-establishes separate intra-cranial and extra-cranial compartments. Defect size is not a restrictive factor as large sizes bone graft (calvarial/nasal septum) or fascia lata graft can be easily harvested. The technique can be easily mastered and does not require a long learning curve. In our series, no patient had recurrent cerebrospinal fluid leak or recurrent herniation of cranial contents on a minimum of 24 months of follow-up period. We had no case of bone graft migration in follow-up studies. It thus seems justifiable to continue to use this technique for repairs of skull base defects. Further prospective evaluation of this novel technique needs to be undertaken.
